# Supplementary material for: A Critical View of the Application of the APEX Software (Aqueous Photochemistry of Environmentally-Occurring Xenobiotics) to Predict Photoreaction Kinetics in Surface Freshwaters
Source: Molecules. 2019 Dec 18;25(1):9. doi: 10.3390/molecules25010009 (PMC7017383; doi:10.3390/molecules25010009)
Supplement: Supplementary file 1 [file molecules-25-00009-s001.zip › 3.2.4_gcc-4.4.0.zip!/3.2.4_gcc-4.4.0/3.2.4_gcc-4.4.0/share/octave/packages/io-1.0.11/doc/READ-ODS.html]

**ODS
support for Octave**

Copyright
© 2009, 2010 Philip Nienhuis <prnienhuis at users.sf.net>

This
version March 1, 2010

*(ODS
= Open Document Format spreadsheet data format, used by e.g.,
OpenOffice.org.)*

**Files
content**

***odsread.m***

No-hassle
read script for reading from an ODS file and parsing the numeric and
text data into separate arrays.

***odswrite.m***

No-hassle
write script for writing to an ODS file.

***odsopen.m***

Get
a file pointer to an ODS spreadsheet file.

***ods2oct.m***

Read
raw data from an ODS spreadsheet file using the file pointer handed
by odsopen.

***oct2ods.m***

Write
data to an ODS spreadsheet file using the file pointer handed by
odsopen.

***odsclose.m***

Close
file handle made by odsopen and -if data have been transfered to a
spreadsheet- save data.

***odsfinfo.m***

Explore
sheet names and optionally estimated data size of ods files with
unknown content.

***calccelladdress.m***

Utility
function needed for jOpenDocument class.

***parsecell.m***

(contained
in Excel xlsread scripts, but works also for ods support) parse raw
data (cell array) into separate numeric array and text (cell) array.)

**REQUIRED
SUPPORT SOFTWARE**

For
Windows (MingW):

- octave
  with java package (>= 1.2.6)

For
Linux:

- octave
  with java package (>= 1.2.5; earlier versions not tested)

For
ODS access, you'll need to choose at least one of the following java
class files collections:

- (currently
  the preferred option) **odfdom.jar** (only version 0.7.5 works
  OK!) & **xercesImpl.jar**. Get them here:

  - http://odftoolkit.org/projects/odfdom/downloads/directory/previous-versions%252Freleases

    - http://xerces.apache.org/mirrors.cgi

and/or

- **jopendocument**<version>**.jar**.
  Get it from http://www.jopendocument.org

These
must be referenced with full pathnames in your javaclasspath. Hint:
add it in ./share/octave/<version>/m/startup/octaverc using
appropriate javaaddpath statements.

**USAGE**

(see
“help ods<function\_filename>” in octave terminal.)

**odsread**
is
a sort of analog to xlsread and works more or less the same. **odsread** is
a mere wrapper for the functions **odsopen**,**ods2oct,**
and
**odsclos**e
that do file access and the actual reading, plus **parsecell**
for
post-processing.

**odswrite**
works
similar to xlswrite. It too is a wrapper for scripts which do the
actual work and invoke other scripts.

**odsfinfo**
can
be used to explore odsfiles with unknown content for sheet names and
to get an impression of the data content sizes.

When
you need data from just one sheet, odsread is for you.

But
when you need data from multiple sheets in the same spreadsheet file,
or if you want to process spreadsheet data by limited-size chunks at
a time, odsopen / ods2oct [/parsecell] / … / odsclose
sequences provides for much more speed and flexibility as the
spreadsheet needs to be read just once rather than repeatedly for
each call to odsread.

Same
reasoning goes for odswrite.

Also,
if you use odsopen / …../, you can process multiple
spreadsheets simultaneously – just use odsopen repeatedly to
get multiple spreadsheet file pointers.

Moreover,
after adding data to an existing spreadsheet file, you can fiddle
with the filename in the ods file pointer struct to save the data
into another, possibly new spreadsheet file.

If
you use odsopen / ods2oct / … / odsclose, DO NOT FORGET to
invoke odsclose in the end. The file pointers can contain an enormous
amount of data and may needlessly keep precious memory allocated.

**GOTCHAS**

I
know of one big gotcha: i.e. reading dates (& time). A less
obvious one is java memory pool allocation size.

**Date
and time in ODS**

Octave
(as does Matlab) stores dates as a number representing the number of
days since January 1, 0 (and as an aside ignores a.o. Pope Gregorius'
intervention in 1582 when 10 days were simply skipped).

OpenOffice.org
stores dates as text strings like “yyyy-mm-dd”.

MS-Excel
stores dates as a number representing the number of days since
January 1, 1900 (and as an aside, erroneously assumes 1900 to be a
leap year).

Now,
converting OpenOffice.org date cell values into Octave looks pretty
straightforward. But when the ODS spreadsheet was originally an Excel
spreadsheet converted by OpenOffice.org, the date cells can either be
OOo date values (i.e.,strings) OR old numerical values from the Excel
spreadsheet.

So:
you should carefully check what happens to date cells.

As
octave has no ”date” or “time” data type,
octave date values (usually numerical data) are simply transferred as
“floats” to ODS spreadsheets. You'll have to convert the
values into dates yourself from within OpenOffice.org.

While
adding data and time values has been implemented in the write
scripts, the wait is for clever solutions to distinguish dates from
floats in octave cell arrays.

**Java
memory pool allocation size**

The
java virtual machine (JVM) initializes one big chunk of your
computer's RAM in which all java classes and methods etc. are to be
loaded: the java memory pool. It does this because java has a very
sophisticated “garbage collection” system. At least on
Windows, the initial size is 2MB and the maximum size is 64MB. On
Linux this allocated size is much bigger. This part of memory is
where the java-based ODS octave routines (and the java-based xls
routines) live and keep their variables etc.

For
transferring large pieces of information to and from spreadsheets you
might hit the limits of this pool. E.g. to be able to handle I/O of
an array of around 50,000 cells I needed a memory pool size of 512
MB.

The
memory size can be increased by inserting a file called “java.opts”
(without quotes) in the directory
./share/octave/packages/java-<version> (where the script file
javaclasspath.m is located), containing just the following lines:

**-Xms16m**

**-Xmx512m**

(where
16 = initial size, 512 = maximum size, m stands for Megabyte).

After
processing a large chunk of spreadsheet information you might notice
that octave's memory footprint does not shrink so it looks like
java's memory pool does not shrink back; but rest assured, the memory
footprint is the *allocated* (reserved)
memory size, not the actual used size. After the JVM has done its
garbage collection, only the so-called “working set” of
the memory allocation is really in use and that is a trimmed-down
part of the memory allocation pool. On Windows systems it often
suffices to minimize the octave terminal for a few seconds to get a
more reasonable memory footprint.

Smaller
gotcha's (only with jOpenDocument):

- While
  reading, empty cells are sometimes not skipped but interpreted with
  numerical value 0 (zero).

  - A
    valid range MUST be specified, I haven't found a way to discover the
    actual occupied rows and columns (jOpenDocument can give the
    physical ones (= capacity) but that doesn't help).

**MATLAB
COMPATIBILITY**

AFAIK
there's no similar functionality in Matlab (yet?).

odsread
is fairly function-compatible to xlsread, however.

Same
goes for odswrite, odsfinfo and xlsfinfo – however odsinfo has
better functionality IMO.

**COMPARISON
OF INTERFACES**

The
ODFtoolkit (& associated xerces) interface is the one that gives
the best (but slow) results at present. However, parsing xml trees
into rectangular arrays is not quite straightforward and the other
way round is a real nightmare; odftoolkit does little to hide the
gory details for the developers.

While
reading ODS is still OK, writing implies checking whether cells
already exist explicitly (in table:table-cells) or implicitly (in
number-columns-repeated or number-rows-repeated nodes) or not at all
yet in which case you'll need to add various types of parent nodes.
Inserting new cells (“nodes”) or deleting nodes implies
rebuilding possibly large parts of the tree in memory - nothing for
the faint-of-heart. And odftoolkit lets you sort it out all by
yourself.

The
jOpenDocument interface is the most promising, as it does shield the
xml tree details and presents developers something which looks like a
spreadsheet model.

However,
unfortunately the developers decided to shield essential methods by
making them 'protected' (e.g. the vital getCellType). Extracting
sheet names is not implemented in released versions (yet).

JopenDocument
does support writing, however I couldn't reliably create new
MutableCells beyond column 1 no matter how hard I tried. The
developers gave me hints but I haven't found a final solution yet.

And
last (but not least) the jOpenDocument developers state that their
development is primarily driven by requests from customers who pay
for support. I do sympathize with this business model but for octave
needs this may hamper progress for a while.

**DEVELOPMENT**

As
with the Excel r/w stuff, adding new interfaces should be easy and
straightforward.

Suggestions
for future development:

- Reliable
  and easy ODS write support (maybe when jOpenDocument is more mature)

  - Speeding
    up (ODS is 10 X slower than e.g. OOXML !!!). jOpenDocument is
    muchfasterbut

    - “Passing
      function handle” a la Matlab's xlsread.

Enjoy!

Philip
Nienhuis, Mar 01, 2010
